# 2-[(2-Carboxyphenyl)disulfanyl]benzoate (1,5-dimethyl-3-oxo-2-phenyl-2,3-di­hydro-1*H*-pyrazol-4-yl)ammonium

**DOI:** 10.1107/S1600536809036952

**Published:** 2009-10-10

**Authors:** Jian-Zhong Huo

**Affiliations:** a College of Chemistry and Life Science, Tianjin Key Laboratory of Structure and Performance for Functional Molecules, Tianjin Normal University, Tianjin 300387, People’s Republic of China

## Abstract

In the title molecular salt, C_11_H_14_N_3_O^+^·C_14_H_9_O_4_S_2_^−^, one of the carboxylic groups of the 2,2′-dithio­dibenzoic acid is deprotonated and the exocyclic amino N atom of the 4-amino­anti­pyrine is protonated. In the anion, the dihedral angle between the two benzene rings is 73.51 (5)° and in the cation the dihedral angle between the phenyl ring and the five-membered ring is 65.79 (9)°. In the crystal structure, inter­molecular N—H⋯O and O—H⋯O hydrogen bonds connect the anions and cations into chains along [010].

## Related literature

For mol­ecular recognition by intermolecular non-covalent interactions, see Rebek (1990 ▶); Remenar *et al.* (2003 ▶). For the properties and applications of 4-amino­anti­pyrine and its derivatives, see Wang *et al.* (2008*b* ▶); Ismail *et al.* (1997 ▶); Selvakumar *et al.* (2007 ▶); Meffin *et al.* (1977 ▶). For the structures and properties of 2,2′-dithio­dibenzoic acid-based metal complexes and cocrystals, see Basiuk *et al.* (1999 ▶); Murugavel *et al.* (2001 ▶); Broker *et al.* (2007 ▶; 2008 ▶); Meng *et al.* (2008 ▶); Wang *et al*, (2008*a* ▶, 2009 ▶).
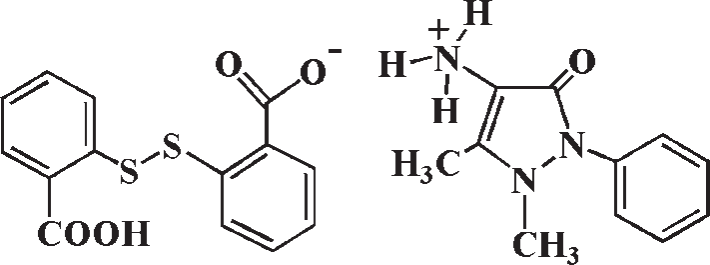


## Experimental

### 

#### Crystal data

C_11_H_14_N_3_O^+^·C_14_H_9_O_4_S_2_^−^
*M* *_r_* = 509.58
Triclinic, 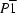

*a* = 9.661 (3) Å
*b* = 10.283 (4) Å
*c* = 13.829 (7) Å
α = 99.872 (7)°
β = 91.929 (7)°
γ = 115.244 (5)°
*V* = 1215.5 (9) Å^3^
*Z* = 2
Mo *K*α radiation
μ = 0.26 mm^−1^
*T* = 296 K
0.30 × 0.28 × 0.22 mm

#### Data collection

Brucker APEXII CCD area-detector diffractometer
Absorption correction: multi-scan (*SADABS*; Sheldrick, 1996 ▶) *T*_min_ = 0.926, *T*_max_ = 0.945
6173 measured reflections
4227 independent reflections
3634 reflections with *I* > 2σ(*I*)
*R*_int_ = 0.012

#### Refinement

*R*[*F* ^2^ > 2σ(*F* ^2^)] = 0.036
*wR*(*F* ^2^) = 0.100
*S* = 1.06
4227 reflections
320 parameters
H-atom parameters constrained
Δρ_max_ = 0.20 e Å^−3^
Δρ_min_ = −0.23 e Å^−3^

### 

Data collection: *APEX2* (Bruker, 2003 ▶); cell refinement: *SAINT* (Bruker, 2001 ▶); data reduction: *SAINT*; program(s) used to solve structure: *SHELXS97* (Sheldrick, 2008 ▶); program(s) used to refine structure: *SHELXL97* (Sheldrick, 2008 ▶); molecular graphics: *SHELXTL* (Sheldrick, 2008 ▶) and *DIAMOND* (Brandenburg & Berndt, 1999 ▶); software used to prepare material for publication: *SHELXTL*.

## Supplementary Material

Crystal structure: contains datablocks I, global. DOI: 10.1107/S1600536809036952/lh2900sup1.cif

Structure factors: contains datablocks I. DOI: 10.1107/S1600536809036952/lh2900Isup2.hkl

Additional supplementary materials:  crystallographic information; 3D view; checkCIF report

## Figures and Tables

**Table 1:** Hydrogen-bond geometry (Å, °)

*D*—H⋯*A*	*D*—H	H⋯*A*	*D*⋯*A*	*D*—H⋯*A*
O5—H5⋯O1^i^	0.82	1.72	2.527 (2)	166
N3—H3*A*⋯O3^ii^	0.89	1.68	2.561 (2)	170
N3—H3*B*⋯O4^iii^	0.89	2.00	2.865 (2)	165
N3—H3*C*⋯O2	0.89	1.98	2.834 (2)	161

Symmetry codes: (i) 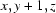; (ii) 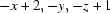; (iii) 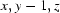.

**Fig. 1.**
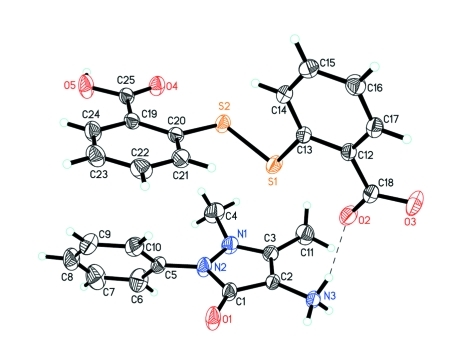
Molecular structure of I. Displacement ellipsoids are drawn at the 30% probability leveral. The dashed line indicates a hydrogen bond.

**Fig. 2.**
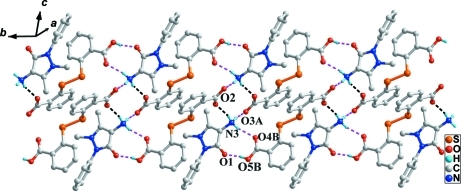
A single hydrogen-bonded one-dimensional ribbon of I [Symmetry codes: (A) 2–x, –y, 1–z; (B) x, -1 + y, z]. Hydrogen bonds are shown as dashed lines.

**Fig. 3.**
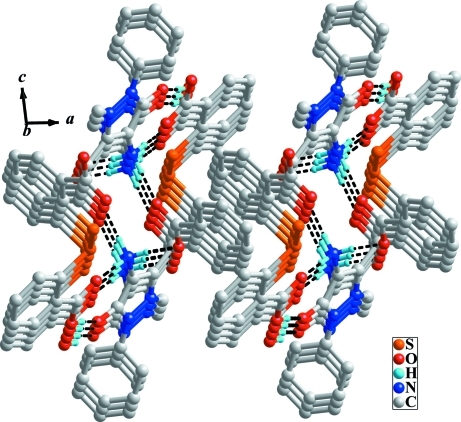
Part of the crystal structure of I with hydrogen bonds shown as dashed lines.

## supplementary crystallographic information

### Comment

Molecular recognition by intermolecular non-covalent interactions such as hydrogen-bonding, π···π stacking and electrostatic interactions, has been receiving more and more attention in diverse research fields (Rebek, 1990). The hydrogen-bonded adducts of active pharmaceutical ingredients with small molecules have rapidly becoming one of intense interest in medicine and crystal engineering fields (Remenar *et al.*, 2003).

In this regard, 4-aminoantipyrine (AP) and its versatile Schiff base derivatives have been extensively used in clinical and pharmacological areas to treat various virus diseases (Meffin *et al.*, 1977; Wang *et al.*, 2008*b*; Ismail *et al.*, 1997; Selvakumar *et al.*, 2007). And the active ingredients may be the individual organic molecules or their metal complexes. On the other hand, 2,2'-dithiodibenzoic acid (H_2_L) is one of the multifunctional molecules containing both carboxylic groups and rotational S—S bond, and can be potentially afforded various hydrogen-bonding sites and diverse supramolecular architectures (Broker *et al.*, 2007). In fact, many hydrogen-bonded H_2_L-involved adducts with controllable deprotonation degree and flexible conformations have been obtained by far (Basiuk *et al.*, 1999; Murugavel *et al.*, 2001; Broker *et al.*, 2008; Meng *et al.* 2008; Wang *et al.*, 2008*a*; Wang *et al.*, 2009).

Herein, to fully explore the solid molecular recognition behavior by hydrogen-bonding interactions, the unsubstituted AP and flexible H_2_L components were selected as building blocks for creating a cocrystal. As a result, a 1:1 adduct with proton transfer, (I), was obtained in ethanol medium.

As shown in Fig. 1, the asymmetric unit of (I) comprises one mono-deprotonated H*L*^-^ anion and one protonated HAP^+^ ion for charge balance. The two components in the asymmetric unit are connected by an N3–H3C···O2 hydrogen bond between the exocyclic amino group of HAP^+^ and the deprotonated carboxylate of H*L*^-^. In the cation, the mean plane of the phenyl ring is rotated by 65.79 (9) ° with respect to the five-membered pyrazoline ring. When viewed along the central S–S bond, the H*L*^-^ anion adopts a characteristic *L*-shaped conformation. The torsion angle of C13—S1—S2—C20 is -83.03 (1)°, and the dihedral angle between the two benzene rings is 73.51 (5)Å. The carboxylic residues of H*L*^-^ are essentially co-planar with respect to the benzene rings.

In the crystal structure of I, two hydrogen-bonded dimers from the adjacent asymmetric units are further aggregated together by a pair of N3–H3A··· O3 interactions, leading to the formation of the centro-symmetric tetramer. The tetramers are then extended along the crystallographic *b*-axis by N3–H3B··· O4 and O5–H5··· O1 recognition patterns (Table 1). As a result, an extended ribbon-like supramolecular assembly was obtained (Fig. 2). There are no interactions between  adjacent ribbons. (Fig. 3).

### Experimental

To a hot ethanol solution (5 ml) containing 2,2'-dithiodibenzoic acid (15.3 mg, 0.05 mmol) was slowly added an ethanol solution (5 ml) of 4-aminoantipyrine (20.3 mg 0.1 mmol) with constant stirring. The resulting mixture was further heated for one hour and then filtered. Upon slow evaporation of the filtrate at room temperature, yellow block-shaped crystals suitable for X-ray analysis were generated within ten days in 60% yield (based on 2,2'-dithiodibenzoic acid). Elemental analysis calculated for C_25_H_23_N_3_O_5_S_2:_ C, 58.92; H, 4.55; N, 8.25%; found: C, 58.93; H, 4.58; N, 8.35%.

### Refinement

H atoms were located in difference maps, but were subsequently placed in calculated positions and treated as riding, with C—H = 0.93 (aromatic) or 0.96 (methyl), O—H = 0.82, and N—H = 0.89 Å. All H-atoms were allocated displacement parameters related to those parent atoms [*U*_iso_(H)= 1.2 *U*_eq_ (C) or *U*_iso_(H)= 1.5 *U*_eq_ (C_methyl_,N,O) ].

### Crystal data

**Table:**

C_11_H_14_N_3_O^+^·C_14_H_9_O_4_S_2_^−^	*Z* = 2
*M**_r_* = 509.58	*F*(000) = 532
Triclinic, *P*1	*D*_x_ = 1.392 Mg m^−^^3^
*a* = 9.661 (3) Å	Mo *K*α radiation, λ = 0.71073 Å
*b* = 10.283 (4) Å	Cell parameters from 4106 reflections
*c* = 13.829 (7) Å	θ = 2.4–27.8°
α = 99.872 (7)°	µ = 0.26 mm^−^^1^
β = 91.929 (7)°	*T* = 296 K
γ = 115.244 (5)°	Block, yellow
*V* = 1215.5 (9) Å^3^	0.30 × 0.28 × 0.22 mm

### Data collection

**Table:**

Brucker APEXII CCD area-detector diffractometer	4227 independent reflections
Radiation source: fine-focus sealed tube	3634 reflections with *I* > 2σ(*I*)
graphite	*R*_int_ = 0.012
phi and ω scans	θ_max_ = 25.0°, θ_min_ = 1.5°
Absorption correction: multi-scan (*SADABS*; Sheldrick, 1996)	*h* = −11→11
*T*_min_ = 0.926, *T*_max_ = 0.945	*k* = −5→12
6173 measured reflections	*l* = −16→15

### Refinement

**Table:**

Refinement on *F*^2^	Primary atom site location: structure-invariant direct methods
Least-squares matrix: full	Secondary atom site location: difference Fourier map
*R*[*F*^2^ > 2σ(*F*^2^)] = 0.036	Hydrogen site location: inferred from neighbouring sites
*wR*(*F*^2^) = 0.100	H-atom parameters constrained
*S* = 1.06	*w* = 1/[σ^2^(*F*_o_^2^) + (0.0499*P*)^2^ + 0.3886*P*] where *P* = (*F*_o_^2^ + 2*F*_c_^2^)/3
4227 reflections	(Δ/σ)_max_ < 0.001
320 parameters	Δρ_max_ = 0.20 e Å^−^^3^
0 restraints	Δρ_min_ = −0.23 e Å^−^^3^

### Special details

**Table:**

Geometry. All e.s.d.'s (except the e.s.d. in the dihedral angle between two l.s. planes) are estimated using the full covariance matrix. The cell e.s.d.'s are taken into account individually in the estimation of e.s.d.'s in distances, angles and torsion angles; correlations between e.s.d.'s in cell parameters are only used when they are defined by crystal symmetry. An approximate (isotropic) treatment of cell e.s.d.'s is used for estimating e.s.d.'s involving l.s. planes.
Refinement. Refinement of *F*^2^ against ALL reflections. The weighted *R*-factor *wR* and goodness of fit *S* are based on *F*^2^, conventional *R*-factors *R* are based on *F*, with *F* set to zero for negative *F*^2^. The threshold expression of *F*^2^ > σ(*F*^2^) is used only for calculating *R*-factors(gt) *etc*. and is not relevant to the choice of reflections for refinement. *R*-factors based on *F*^2^ are statistically about twice as large as those based on *F*, and *R*- factors based on ALL data will be even larger.

### Fractional atomic coordinates and isotropic or equivalent isotropic displacement parameters (Å^2^)

**Table:**

	*x*	*y*	*z*	*U*_iso_*/*U*_eq_	
S1	0.79625 (6)	0.30246 (6)	0.41198 (4)	0.04987 (15)	
S2	0.77232 (6)	0.46456 (5)	0.35447 (3)	0.04581 (14)	
O2	0.87236 (18)	0.12722 (17)	0.49915 (11)	0.0596 (4)	
O3	0.78833 (18)	0.04135 (19)	0.63321 (12)	0.0653 (4)	
O4	0.78550 (16)	0.67278 (14)	0.25767 (10)	0.0518 (3)	
O5	0.6376 (2)	0.65050 (16)	0.12387 (11)	0.0703 (5)	
H5	0.6937	0.7388	0.1319	0.105*	
C12	0.6718 (2)	0.17200 (19)	0.56767 (12)	0.0379 (4)	
C13	0.6661 (2)	0.26280 (19)	0.50372 (12)	0.0385 (4)	
C14	0.5566 (2)	0.3168 (2)	0.51215 (14)	0.0450 (4)	
H14	0.5522	0.3773	0.4705	0.054*	
C15	0.4543 (2)	0.2822 (2)	0.58140 (15)	0.0516 (5)	
H15	0.3816	0.3192	0.5859	0.062*	
C16	0.4590 (2)	0.1929 (2)	0.64402 (15)	0.0523 (5)	
H16	0.3897	0.1693	0.6906	0.063*	
C17	0.5674 (2)	0.1390 (2)	0.63682 (14)	0.0452 (4)	
H17	0.5708	0.0791	0.6793	0.054*	
C18	0.7866 (2)	0.1099 (2)	0.56449 (14)	0.0435 (4)	
C19	0.5772 (2)	0.44117 (19)	0.19213 (12)	0.0411 (4)	
C20	0.6068 (2)	0.36948 (19)	0.26285 (12)	0.0399 (4)	
C21	0.5030 (2)	0.2243 (2)	0.26044 (14)	0.0504 (5)	
H21	0.5192	0.1761	0.3076	0.060*	
C22	0.3767 (3)	0.1507 (2)	0.18949 (16)	0.0577 (5)	
H22	0.3083	0.0542	0.1899	0.069*	
C23	0.3508 (3)	0.2184 (2)	0.11810 (16)	0.0591 (5)	
H23	0.2675	0.1673	0.0690	0.071*	
C24	0.4498 (3)	0.3629 (2)	0.12028 (14)	0.0537 (5)	
H24	0.4314	0.4095	0.0728	0.064*	
C25	0.6773 (2)	0.5980 (2)	0.19429 (13)	0.0432 (4)	
O1	0.7722 (2)	−0.07506 (15)	0.13515 (11)	0.0708 (5)	
N1	1.0500 (2)	0.28242 (17)	0.21502 (13)	0.0522 (4)	
N2	0.9294 (2)	0.17231 (17)	0.14899 (12)	0.0544 (4)	
N3	0.95999 (18)	−0.01942 (17)	0.33782 (11)	0.0420 (4)	
H3A	1.0521	−0.0163	0.3517	0.063*	
H3B	0.8939	−0.1104	0.3082	0.063*	
H3C	0.9266	0.0071	0.3936	0.063*	
C1	0.8789 (2)	0.0433 (2)	0.18279 (14)	0.0479 (5)	
C2	0.9719 (2)	0.07944 (19)	0.27335 (13)	0.0381 (4)	
C3	1.0727 (2)	0.2242 (2)	0.29096 (14)	0.0451 (4)	
C4	1.1063 (3)	0.4359 (2)	0.2109 (2)	0.0746 (7)	
H4A	1.0228	0.4630	0.2147	0.112*	
H4B	1.1488	0.4519	0.1499	0.112*	
H4C	1.1847	0.4947	0.2654	0.112*	
C5	0.8733 (2)	0.1957 (2)	0.05991 (14)	0.0473 (5)	
C6	0.9637 (3)	0.2291 (3)	−0.01490 (19)	0.0736 (7)	
H6	1.0623	0.2347	−0.0089	0.088*	
C7	0.9072 (4)	0.2545 (3)	−0.09933 (19)	0.0868 (9)	
H7	0.9685	0.2780	−0.1501	0.104*	
C8	0.7649 (4)	0.2455 (3)	−0.10844 (19)	0.0796 (8)	
H8	0.7295	0.2661	−0.1646	0.096*	
C9	0.6711 (4)	0.2065 (3)	−0.0363 (2)	0.0816 (8)	
H9	0.5709	0.1966	−0.0449	0.098*	
C10	0.7251 (3)	0.1817 (3)	0.04980 (17)	0.0643 (6)	
H10	0.6623	0.1562	0.0996	0.077*	
C11	1.1906 (3)	0.3128 (3)	0.37761 (18)	0.0691 (6)	
H11A	1.2875	0.3677	0.3551	0.104*	
H11B	1.2017	0.2486	0.4169	0.104*	
H11C	1.1586	0.3793	0.4168	0.104*	

### Atomic displacement parameters (Å^2^)

**Table:**

	*U*^11^	*U*^22^	*U*^33^	*U*^12^	*U*^13^	*U*^23^
S1	0.0581 (3)	0.0600 (3)	0.0518 (3)	0.0376 (3)	0.0180 (2)	0.0287 (2)
S2	0.0526 (3)	0.0423 (3)	0.0456 (3)	0.0208 (2)	0.0031 (2)	0.0176 (2)
O2	0.0725 (10)	0.0751 (10)	0.0613 (9)	0.0521 (8)	0.0246 (8)	0.0331 (8)
O3	0.0657 (9)	0.0869 (11)	0.0742 (10)	0.0483 (9)	0.0208 (8)	0.0522 (9)
O4	0.0579 (8)	0.0390 (7)	0.0534 (8)	0.0144 (6)	−0.0025 (7)	0.0175 (6)
O5	0.1014 (13)	0.0418 (8)	0.0523 (8)	0.0151 (8)	−0.0180 (8)	0.0205 (7)
C12	0.0403 (9)	0.0369 (9)	0.0357 (9)	0.0164 (8)	−0.0008 (7)	0.0082 (7)
C13	0.0435 (9)	0.0384 (9)	0.0352 (9)	0.0197 (8)	0.0010 (7)	0.0081 (7)
C14	0.0530 (11)	0.0489 (11)	0.0423 (10)	0.0299 (9)	0.0030 (8)	0.0128 (8)
C15	0.0510 (11)	0.0622 (13)	0.0517 (11)	0.0343 (10)	0.0082 (9)	0.0115 (10)
C16	0.0507 (11)	0.0627 (13)	0.0483 (11)	0.0273 (10)	0.0135 (9)	0.0159 (10)
C17	0.0496 (11)	0.0457 (10)	0.0424 (10)	0.0204 (9)	0.0045 (8)	0.0158 (8)
C18	0.0467 (10)	0.0448 (10)	0.0442 (10)	0.0230 (9)	0.0022 (8)	0.0148 (8)
C19	0.0538 (11)	0.0369 (9)	0.0336 (9)	0.0197 (8)	0.0073 (8)	0.0099 (7)
C20	0.0527 (10)	0.0356 (9)	0.0341 (9)	0.0210 (8)	0.0085 (8)	0.0092 (7)
C21	0.0682 (13)	0.0366 (10)	0.0459 (10)	0.0204 (9)	0.0075 (9)	0.0143 (8)
C22	0.0692 (14)	0.0354 (10)	0.0555 (12)	0.0116 (10)	0.0069 (10)	0.0074 (9)
C23	0.0633 (13)	0.0485 (12)	0.0496 (12)	0.0130 (10)	−0.0064 (10)	0.0035 (10)
C24	0.0676 (13)	0.0500 (12)	0.0406 (10)	0.0222 (10)	−0.0005 (9)	0.0134 (9)
C25	0.0579 (12)	0.0404 (10)	0.0343 (9)	0.0219 (9)	0.0082 (9)	0.0136 (8)
O1	0.0899 (11)	0.0384 (8)	0.0604 (9)	0.0052 (8)	−0.0302 (8)	0.0196 (7)
N1	0.0551 (10)	0.0353 (8)	0.0576 (10)	0.0109 (7)	−0.0019 (8)	0.0138 (7)
N2	0.0644 (11)	0.0375 (9)	0.0512 (9)	0.0111 (8)	−0.0097 (8)	0.0177 (7)
N3	0.0460 (8)	0.0442 (8)	0.0392 (8)	0.0217 (7)	0.0008 (6)	0.0134 (7)
C1	0.0586 (12)	0.0371 (10)	0.0438 (10)	0.0152 (9)	−0.0030 (9)	0.0153 (8)
C2	0.0412 (9)	0.0383 (9)	0.0375 (9)	0.0188 (8)	0.0030 (7)	0.0110 (7)
C3	0.0457 (10)	0.0430 (10)	0.0463 (10)	0.0194 (9)	0.0020 (8)	0.0096 (8)
C4	0.0852 (17)	0.0384 (12)	0.0888 (18)	0.0133 (11)	0.0020 (14)	0.0231 (12)
C5	0.0616 (12)	0.0379 (10)	0.0455 (10)	0.0216 (9)	0.0034 (9)	0.0175 (8)
C6	0.0665 (15)	0.0874 (18)	0.0727 (16)	0.0297 (13)	0.0191 (12)	0.0403 (14)
C7	0.104 (2)	0.086 (2)	0.0589 (15)	0.0217 (17)	0.0183 (15)	0.0382 (14)
C8	0.119 (2)	0.0490 (13)	0.0605 (15)	0.0255 (14)	−0.0183 (15)	0.0213 (11)
C9	0.094 (2)	0.0870 (19)	0.0804 (18)	0.0587 (17)	−0.0129 (15)	0.0139 (15)
C10	0.0746 (15)	0.0760 (16)	0.0574 (13)	0.0460 (13)	0.0114 (11)	0.0161 (11)
C11	0.0650 (14)	0.0538 (13)	0.0676 (15)	0.0113 (11)	−0.0175 (12)	0.0049 (11)

### Geometric parameters (Å, °)

**Table:**

S1—C13	1.7901 (19)	O1—C1	1.260 (2)
S1—S2	2.0580 (9)	N1—C3	1.350 (2)
S2—C20	1.796 (2)	N1—N2	1.387 (2)
O2—C18	1.231 (2)	N1—C4	1.446 (3)
O3—C18	1.280 (2)	N2—C1	1.376 (2)
O4—C25	1.214 (2)	N2—C5	1.427 (2)
O5—C25	1.311 (2)	N3—C2	1.435 (2)
O5—H5	0.8200	N3—H3A	0.8900
C12—C17	1.390 (3)	N3—H3B	0.8900
C12—C13	1.407 (2)	N3—H3C	0.8900
C12—C18	1.496 (3)	C1—C2	1.412 (3)
C13—C14	1.389 (3)	C2—C3	1.359 (3)
C14—C15	1.379 (3)	C3—C11	1.487 (3)
C14—H14	0.9300	C4—H4A	0.9600
C15—C16	1.378 (3)	C4—H4B	0.9600
C15—H15	0.9300	C4—H4C	0.9600
C16—C17	1.377 (3)	C5—C6	1.370 (3)
C16—H16	0.9300	C5—C10	1.377 (3)
C17—H17	0.9300	C6—C7	1.383 (4)
C19—C24	1.396 (3)	C6—H6	0.9300
C19—C20	1.410 (2)	C7—C8	1.337 (4)
C19—C25	1.479 (3)	C7—H7	0.9300
C20—C21	1.393 (3)	C8—C9	1.365 (4)
C21—C22	1.379 (3)	C8—H8	0.9300
C21—H21	0.9300	C9—C10	1.390 (3)
C22—C23	1.375 (3)	C9—H9	0.9300
C22—H22	0.9300	C10—H10	0.9300
C23—C24	1.376 (3)	C11—H11A	0.9600
C23—H23	0.9300	C11—H11B	0.9600
C24—H24	0.9300	C11—H11C	0.9600
			
C13—S1—S2	105.53 (6)	C1—N2—N1	109.44 (15)
C20—S2—S1	105.13 (6)	C1—N2—C5	127.62 (16)
C25—O5—H5	109.5	N1—N2—C5	122.93 (15)
C17—C12—C13	118.95 (17)	C2—N3—H3A	109.5
C17—C12—C18	118.54 (16)	C2—N3—H3B	109.5
C13—C12—C18	122.51 (16)	H3A—N3—H3B	109.5
C14—C13—C12	118.82 (16)	C2—N3—H3C	109.5
C14—C13—S1	121.61 (14)	H3A—N3—H3C	109.5
C12—C13—S1	119.55 (13)	H3B—N3—H3C	109.5
C15—C14—C13	121.01 (17)	O1—C1—N2	122.29 (17)
C15—C14—H14	119.5	O1—C1—C2	132.74 (17)
C13—C14—H14	119.5	N2—C1—C2	104.97 (16)
C16—C15—C14	120.43 (18)	C3—C2—C1	109.02 (16)
C16—C15—H15	119.8	C3—C2—N3	125.26 (16)
C14—C15—H15	119.8	C1—C2—N3	125.71 (16)
C17—C16—C15	119.23 (18)	N1—C3—C2	108.82 (16)
C17—C16—H16	120.4	N1—C3—C11	122.47 (18)
C15—C16—H16	120.4	C2—C3—C11	128.71 (18)
C16—C17—C12	121.57 (18)	N1—C4—H4A	109.5
C16—C17—H17	119.2	N1—C4—H4B	109.5
C12—C17—H17	119.2	H4A—C4—H4B	109.5
O2—C18—O3	124.23 (18)	N1—C4—H4C	109.5
O2—C18—C12	120.14 (16)	H4A—C4—H4C	109.5
O3—C18—C12	115.62 (17)	H4B—C4—H4C	109.5
C24—C19—C20	119.15 (17)	C6—C5—C10	120.4 (2)
C24—C19—C25	119.83 (16)	C6—C5—N2	121.0 (2)
C20—C19—C25	121.01 (16)	C10—C5—N2	118.60 (19)
C21—C20—C19	118.20 (17)	C5—C6—C7	119.6 (3)
C21—C20—S2	121.18 (14)	C5—C6—H6	120.2
C19—C20—S2	120.61 (14)	C7—C6—H6	120.2
C22—C21—C20	121.23 (18)	C8—C7—C6	120.4 (3)
C22—C21—H21	119.4	C8—C7—H7	119.8
C20—C21—H21	119.4	C6—C7—H7	119.8
C23—C22—C21	120.71 (19)	C7—C8—C9	120.8 (2)
C23—C22—H22	119.6	C7—C8—H8	119.6
C21—C22—H22	119.6	C9—C8—H8	119.6
C22—C23—C24	119.10 (19)	C8—C9—C10	120.1 (3)
C22—C23—H23	120.4	C8—C9—H9	119.9
C24—C23—H23	120.4	C10—C9—H9	119.9
C23—C24—C19	121.53 (19)	C5—C10—C9	118.6 (2)
C23—C24—H24	119.2	C5—C10—H10	120.7
C19—C24—H24	119.2	C9—C10—H10	120.7
O4—C25—O5	122.76 (17)	C3—C11—H11A	109.5
O4—C25—C19	122.53 (16)	C3—C11—H11B	109.5
O5—C25—C19	114.70 (17)	H11A—C11—H11B	109.5
C3—N1—N2	107.74 (15)	C3—C11—H11C	109.5
C3—N1—C4	128.07 (19)	H11A—C11—H11C	109.5
N2—N1—C4	122.29 (18)	H11B—C11—H11C	109.5
			
C13—S1—S2—C20	−83.01 (9)	C20—C19—C25—O5	179.46 (17)
C17—C12—C13—C14	0.2 (3)	C3—N1—N2—C1	−1.6 (2)
C18—C12—C13—C14	−179.47 (17)	C4—N1—N2—C1	−167.0 (2)
C17—C12—C13—S1	−178.34 (13)	C3—N1—N2—C5	179.52 (19)
C18—C12—C13—S1	2.0 (2)	C4—N1—N2—C5	14.1 (3)
S2—S1—C13—C14	11.17 (17)	N1—N2—C1—O1	−178.3 (2)
S2—S1—C13—C12	−170.30 (13)	C5—N2—C1—O1	0.6 (4)
C12—C13—C14—C15	−0.3 (3)	N1—N2—C1—C2	1.1 (2)
S1—C13—C14—C15	178.23 (15)	C5—N2—C1—C2	179.9 (2)
C13—C14—C15—C16	0.1 (3)	O1—C1—C2—C3	179.0 (2)
C14—C15—C16—C17	0.2 (3)	N2—C1—C2—C3	−0.2 (2)
C15—C16—C17—C12	−0.3 (3)	O1—C1—C2—N3	−2.3 (4)
C13—C12—C17—C16	0.0 (3)	N2—C1—C2—N3	178.49 (17)
C18—C12—C17—C16	179.76 (18)	N2—N1—C3—C2	1.4 (2)
C17—C12—C18—O2	174.18 (18)	C4—N1—C3—C2	165.8 (2)
C13—C12—C18—O2	−6.1 (3)	N2—N1—C3—C11	−177.9 (2)
C17—C12—C18—O3	−6.3 (3)	C4—N1—C3—C11	−13.6 (3)
C13—C12—C18—O3	173.36 (17)	C1—C2—C3—N1	−0.8 (2)
C24—C19—C20—C21	−2.7 (3)	N3—C2—C3—N1	−179.45 (17)
C25—C19—C20—C21	176.19 (17)	C1—C2—C3—C11	178.5 (2)
C24—C19—C20—S2	178.30 (15)	N3—C2—C3—C11	−0.2 (3)
C25—C19—C20—S2	−2.9 (2)	C1—N2—C5—C6	−113.0 (3)
S1—S2—C20—C21	16.17 (17)	N1—N2—C5—C6	65.7 (3)
S1—S2—C20—C19	−164.81 (13)	C1—N2—C5—C10	66.0 (3)
C19—C20—C21—C22	1.6 (3)	N1—N2—C5—C10	−115.3 (2)
S2—C20—C21—C22	−179.32 (16)	C10—C5—C6—C7	2.5 (4)
C20—C21—C22—C23	0.8 (3)	N2—C5—C6—C7	−178.5 (2)
C21—C22—C23—C24	−2.3 (4)	C5—C6—C7—C8	−0.5 (4)
C22—C23—C24—C19	1.2 (3)	C6—C7—C8—C9	−2.2 (4)
C20—C19—C24—C23	1.3 (3)	C7—C8—C9—C10	2.8 (4)
C25—C19—C24—C23	−177.6 (2)	C6—C5—C10—C9	−1.9 (4)
C24—C19—C25—O4	176.68 (19)	N2—C5—C10—C9	179.1 (2)
C20—C19—C25—O4	−2.2 (3)	C8—C9—C10—C5	−0.7 (4)
C24—C19—C25—O5	−1.7 (3)		

### Hydrogen-bond geometry (Å, °)

**Table:**

*D*—H···*A*	*D*—H	H···*A*	*D*···*A*	*D*—H···*A*
O5—H5···O1^i^	0.82	1.72	2.527 (2)	166
N3—H3A···O3^ii^	0.89	1.68	2.561 (2)	170
N3—H3B···O4^iii^	0.89	2.00	2.865 (2)	165
N3—H3C···O2	0.89	1.98	2.834 (2)	161

Symmetry codes: (i) *x*, *y*+1, *z*; (ii) −*x*+2, −*y*, −*z*+1; (iii) *x*, *y*−1, *z*.
